# Novel polymorphisms of dopa decarboxylase gene and their association with lamb quality traits in Indonesian sheep

**DOI:** 10.5713/ab.22.0227

**Published:** 2022-11-14

**Authors:** Ratna Sholatia Harahap, Ronny Rachman Noor, Yuni Cahya Endrawati, Huda Shalahudin Darusman, Asep Gunawan

**Affiliations:** 1 Graduate School of Animal Production and Technology, Faculty of Animal Science, IPB University, Kampus IPB Darmaga Bogor 16680, Indonesia; 2 Department of Animal Production and Technology, Faculty of Animal Science, IPB University, Kampus IPB Darmaga Bogor 16680, Indonesia; 3 Department of Anatomy Physiology and Pharmacology, Faculty of Veterinary Medicine, IPB University, Kampus IPB Darmaga Bogor 16680, Indonesia

**Keywords:** Carcass Characteristics, Dopa Decarboxylase (*DDC*) Gene, Fatty Acid Composition, Lamb Quality, Retail Meat Cut

## Abstract

**Objective:**

This study aimed to investigate the polymorphisms of the dopa decarboxylase (*DDC*) gene and association analysis with lamb quality and expression quantification of the *DDC* gene in phenotypically divergent Indonesian sheep.

**Methods:**

The totals of 189 rams with an average body weight of 24.12 kg at 10 to 12 months were used to identify *DDC* gene polymorphism using polymerase chain reaction-restriction fragment length polymorphism (PCR-RFLP). Among 189 rams, several rams representing various sheep genotypes were used for an association study between genotypes and phenotypic traits with proc general linear model (GLM) analysis. In addition, the gene expression analysis of the DDC mRNA in the phenotypically divergent sheep population was analyzed using quantitative reverse-transcription PCR.

**Results:**

The *DDC* gene (g. 5377439 G>A) showed polymorphisms that indicated three genotypes: AA, AG, and GG. The *DDC* gene polymorphism was significantly associated (p≤0.05) with carcass characteristics including carcass percentage, carcass length, hot and cold carcass; physical properties of lamb quality including pH value; retail cut carcass; fatty acid composition such as fat content, pentadecanoic acid (C15:0), tricosylic acid (C23:0), lignoceric acid (C24:0), oleic acid (C18:1n9c), elaidic acid (C18:1n9t), nervonic acid (C24:1), linoleic acid (C18:2n6c), arachidonic acid (C20:4n6), cervonic acid (C22:6n3); and mineral content including potassium (K). The GG genotype of the *DDC* gene had the best association with lamb quality traits. The *DDC* gene expression analysis mRNA showed no significant difference (p≥0.05) between lamb quality traits.

**Conclusion:**

The *DDC* gene could be used as a potential candidate gene to improve lamb quality.

## INTRODUCTION

Lamb consumption worldwide significantly increased from 2004 to 2014, with a trend that continued into 2022. In Indonesia, lamb consumption has increased in the last five years by 6.56 thousand tons. However, this amount is still insufficient to meet the lamb meat consumption demand, which also recorded an increase of 7.74 thousand tons. As a result, the total consumption of lamb meat in the last year was 135.12 thousand tons [1]. Some of the local sheep that play an essential role in lamb production in Indonesia include Garut sheep (GS), Javanese fat-tailed (JFT), Javanese thin-tailed (JTT), and Jonggol sheep (JS). In addition, some crossbreed sheep are prominent in Indonesia, such as Garut composite sheep (GCS), Compass agrinac sheep (CAS), and Barbados cross sheep (BCS). The effort to increase lamb production is not limited to the number of sheep produced but also to the quality of the lamb.

Lamb quality traits are very complex, involving many factors such as fatty acid composition, lamb tenderness, flavor, and odor [2,3]. Lamb tenderness is influenced by intramuscular fat (IMF) in meat. The amount of IMF and fatty acid composition positively correlated with the meat quality, including sensory properties, healthy considerations, juiciness, tenderness, and flavor compounds [4,5]. Improving lamb quality is expected to increase the meat’s nutritional value, which benefits human health. Therefore, breeding programs are recommended as one of the most realistic approaches, performed using candidate genes through identification variant analysis [6,7]. A few studies by molecular genetics have been done and focused on lamb, including lamb quality traits, carcass characteristics, fatty acid composition, and flavor and odor. As a result, several candidate genes were identified as essential genes influencing the lamb quality [8,9].

The results of transcriptomic studies through RNA Sequencing (RNA-Seq) have identified the functional lamb quality control role of the dopa decarboxylase (*DDC*) gene. The *DDC* gene has been widely reported regarding its role in humans’ brain and nervous system [10–12] and cardiac development [13]. The *DDC* gene is closely related to the production of serotonin in the brain, individuals who are deficient in the *DDC* gene will have decreased serotonin levels leading to developmental delay and movement disorders [11]. In addition, the *DDC* gene encodes one of the enzymes that play an essential role in synthesizing biogenic amines (BA) compounds commonly found in food such as meat. Biogenic amines are a group of molecules playing crucial function in many relevant physiological processes, including nutrition. Therefore, the role of the *DDC* gene in lamb quality is expected to increase lamb’s nutritional value, improving human health [14]. However, information about the *DDC* gene related to characterization and molecular detection is still rare in lamb quality. Therefore, this study aimed to analyze the polymorphism and expression of the *DDC* gene and to describe its association with the lamb quality of Indonesian sheep.

## MATERIALS AND METHODS

### Ethics statement

All experimental procedures, including the care of animals and euthanasia, were approved by the Institutional Animal Care and Use Committee (IACUC) issued by IPB University (approval ID: 117-2018 IPB).

### Animals

The study was performed on 189 rams consisting of 10 Compass agrinac sheep (CAS), 10 Barbados cross sheep (BCS), 10 Garut composite sheep (GCS), 20 Garut sheep (GS), 16 Javanese fat-tail sheep (JFTS), 102 Javanese thin-tail sheep (JTTS), and 21 Jonggol sheep (JS). The CAS, BCS, and GCS were crossbreed sheep, while GS, JFTS, JTTS, and JS were local sheep. The CAS is a crossbreed from local Sumatera (50%), St. Croix sheep (25%), and Barbados black belly sheep (25%). The BCS is a crossbreed from local Sumatera sheep (50%) and Barbados black belly sheep (50%), whereas the GCS are a crossbreed with indigenous Garut sheep (50%), St. Croix sheep (25%), and Moulton Charolais sheep (25%). All rams were maintained under the same management systems in a caged group and fed *ad libitum* with fattening feed. Data for analysis association study with lamb quality was obtained from 150 rams representing various sheep genotypes for association study with carcass characteristics, lamb quality, and fatty acid composition consisted CAS (n = 10), BCS (n = 10), GCS (n = 10), JS (n = 15), JFTS (n = 20), and JTTS (n = 85), while 100 rams for retail cut carcass consisted CAS (n = 10), BCS (n = 10), JS (n = 10), and JTTS (n = 70), and mineral content including JS (n = 15) and JTTS (n = 85). The rams were reared under the same management systems and were fed the same fattening feed with grass and concentrate GTO3.

### Single nucleotide polymorphism selection and slaughter procedure

For the association analysis, the rams were slaughtered in a commercial abattoir PT Pramana Pangan Utama (PPU) Slaughterhouse, at 10–12 months old with an average body weight of 24.12 kg. The single nucleotide polymorphism (SNP) for the present study was selected from the RNA Sequencing result which was located on 3 prime UTR regions and its foundation had a biological function related to bioactive amines. The slaughtering process was carried out based on the Indonesian performance test guidelines. All carcasses were stored at the temperature of 4°C for 24 hours and were cut into two parts: left and right carcasses. The right parts of the carcass were cut into seven pieces of commercial cuts (neck, shoulder, breast-fore shank, rack, loin, flank, and leg) and then divided into meat, bone, and fats (subcutaneous, intramuscular, and pelvic). The longissimus dorsi samples were taken for DNA extraction, analysis of fatty acid, and mineral content analysis. The liver tissues were used for gene expression analysis, and the bicep femoris samples were taken for lamb quality analysis. All pieces were put on ice and stored at −20°C.

### Carcass characteristics and lamb quality analysis

The carcass characteristics measured were live weight, hot and cold weight, length, and carcass percentage. Live weight was measured before the slaughtering process (pre-slaughtered). The hot carcass was weighted immediately after skinning, while the cold carcass was measured after chilling at 4°C for 24 hours. Carcass length was measured from the point of the shoulder to the distal end of the tarsus. The carcass percentage was calculated by the ratio of the hot carcass to the live weight multiplied by 100. Lamb quality traits were analyzed, including pH value, lamb tenderness, cooking loss (CL), and water holding capacity (WHC). The lamb quality-analyzed refers to the method used by Dagong et al [15]. The pH value was measured using a pH meter after storing the carcass for 24 hours post-mortem. Lamb tenderness was measured using Warner Bratzler shear force by measuring the amount of strength (kg/cm^2^) needed to cut the pieces of meat. Cooking loss measurements (%) were calculated as the samples’ weight differences before and after being cooked in a water bath at 80°C for 1 hour. The meat’s WHC was determined by calculating the amount of water loss after being pressured on filter paper for 5 minutes.

### Fatty acid composition analysis

The fatty acid composition was separated and determined using the AOAC 969.333 extraction methods [16]. Then, the fatty acid composition was measured using gas chromatography and expressed as a proportion of the total FAs, including fat content, saturated fatty acid (SFA), monounsaturated fatty acid (MUFA), and polyunsaturated fatty acid (PUFA).

### Mineral content analysis

Mineral content was measured using the IK method LP-04.10-LT-1.0 according to AOAC (2015) Official Method 969.08 (AOAC 4.8.02). The parameters were analyzed, including iron (Fe), zinc (Zn), potassium (K), and selenium (Se).

### DNA extraction and PCR-RFLP amplification

Genomic DNA was extracted from longissimus dorsi using a Genomic DNA Mini Kit (Geneaid Biotech, Taiwan) based on the manufacturing protocol. A pair of specific primers were designed using MEGA 7.0 Software and used to amplify the DDC based on the ovine genome sequence (NCBI accession NC_019461.2; Table 1). The total PCR premix was 15 μL, contained 1 μL of DNA samples, 0.4 μL of primers (forward and reverse), 7.5 μL of MyTaq Red Mix, and 6.1 μL of nucleus water. The amplification process of polymerase chain reaction (PCR) using the AB Systems machine began with the initial denaturation step at 95°C for 1 min, followed by 35 cycles each its, consisting of a denaturation process at 95°C for 15 s, primer annealing at 60°C, and DNA elongation at 72°C for 15 s. The last step was the final elongation at 72°C for 1 min. The DNA amplicon was detected by 1.5 percent agarose gel electrophoresis. The restriction fragment length polymorphism (RFLP) analysis was used for genotyping PCR products [17]. The RFLP could digest PCR amplicons with restriction enzymes to produce distinct polymorphic fragments. The restriction MspI enzymes were used at 37°C for 4 hours. The digested products were separated using a 2% agarose gel and the fragments were visualized under UV Transilluminator (Alpha Imager; Alpha Innotech, Santa Clara, CA, USA).

### *DDC* gene expression analysis in sheep with divergent lamb quality and carcass characteristics

The liver tissue from 12 rams was extracted using the RNeasy Mini Kit (Qiagen, Hilden, Germany) reagent for RNA expression analysis. Three groups, each consisting of four rams, were formed based on AA, AG, and GG genotype. The RNA extract was transcribed into complementary DNA (cDNA) using a First Strand cDNA (Thermo Scientific, Vilnius, Lithuanian, EU) Transcriptor Synthesis kit by reverse transcriptase PCR process. The specific primers for the quantitative reverse-transcription PCR (qRT-PCR) analysis were designed in MEGA 7.0 based on the sequences of ovine DDC and glyceraldehyde-3-phosphate dehydrogenase (GAPDH) (Table 1). Each run examined each cDNA sample and no-template control in a 96-well microtiter plate. The reactions comprised 10 μL, including 5 μL of SYBR Green Master Mix, 0.5 μL of each forward and reverse primers, 2 μL of cDNA (50 ng/μL), and 2 μL of nuclease-free water. All samples were analyzed twice (technical replication), and the geometric mean of the Ct values was further used for mRNA expression profiling. The different plates normalized the target genes using GAPDH and β-Actin housekeeping genes. The qRT-PCR was conducted with the following step: pre-denaturation at 95°C for 30 s, PCR (analysis mode: quantitative) as much 35 cycles at 95°C for 5 s and 60°C for 30 s, melting (analysis mode: melting curve) at 95°C for 5 s, at 60°C for 1 min, and cooling at 50°C for 30 s. The delta Ct (ΔCt) value was calculated as the difference between the cycle threshold (CT) value of the target and the CT value of gene housekeeping [18].

### Data analysis

The polymorphisms of the *DDC* gene were indicated by the level of allele frequency, genotype frequencies, and Hardy-Weinberg equilibrium values calculated according to Lachance [19]. In addition, the association of the *DDC* gene with phenotype was analyzed using a GLM to compare genotypes (Minitab 18 Software) with the following formula:


$${\text{Y}}_{ijk}=\mu +{\text{genotype}}_{i}+{\text{breed}}_{j}+{\text{e}}_{ijk}$$

Where, Y*_ijk_* = the lamb quality, carcass characteristics, retail meat cut, fatty acid composition, and mineral content; μ = the population mean; genotype = the fixed effect of i-th genotype; breed*_j_* = the fixed effect of j-th breed; e*_ijk_* = the residual error.

For *DDC* gene expression analysis, the means of fold change value (ΔCT) per genotype were analyzed as described by Rao et al [18] using calculating the difference between the target gene and the reference gene (ΔCt = Ct_gene target_ – Ct_housekeeping gene_). The phenotype differences for high and low lamb quality and carcass characteristics of *DDC* gene expressions were compared using a T-test by Minitab 18 Software. The significant values (p≤0.05) were considered to show significant differences.

## RESULTS

### Polymorphism of novel gene *DDC*

**Figure 1:**
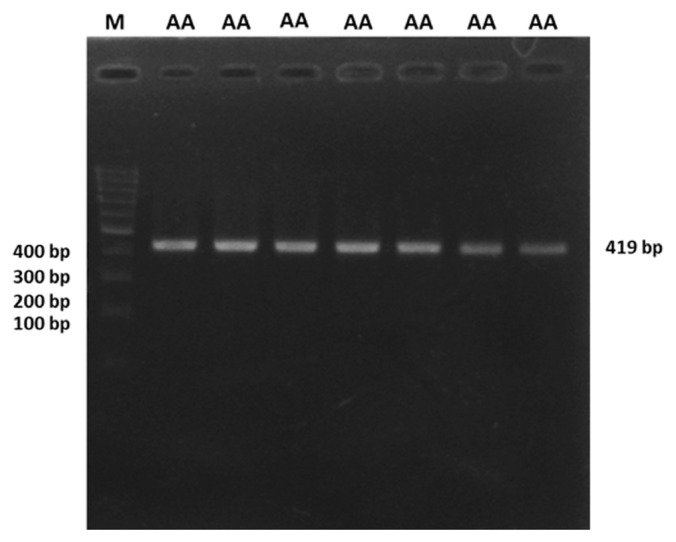
Polymerase chain reaction result for *DDC* gene; M= 100 bp ladder size; AA = genotypes product pcr (419 bp); bp = base pair. *DDC*, dopa decarboxylase.

**Figure 2:**
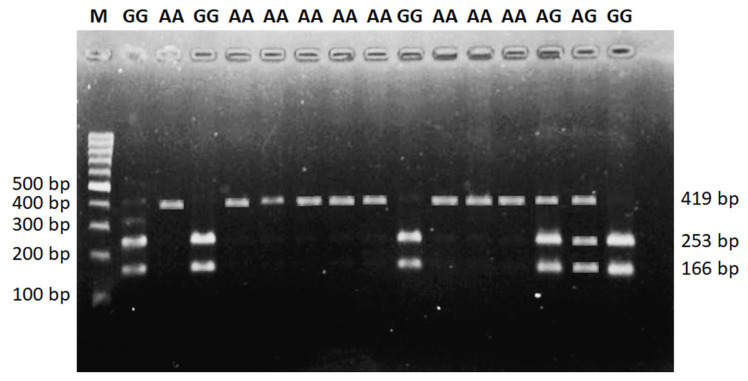
PCR-RFLP genotyping result for *DDC* gene. M = 100 bp ladder size; AA (419 bp), AG (419, 235, and 166 bp), and GG (253 and 166 bp) genotypes; bp = base pair. PCR-RFLP, polymerase chain reaction-restriction fragment length polymorphism; *DDC*, dopa decarboxylase.

A 419 bp by PCR amplicon was obtained, corresponding to the expected size of the *DDC* gene shown in Figure 1. In the study, genotyping of the amplified *DDC* gene target fragments wasconducted by PCR-RFLP analysis. The SNP g. 5377439 G>A of the *DDC* gene polymorphisms were three genotypes, namely GG genotypes (166 and 253 bp), AG (166, 253, 419 bp), and AA genotype (419 bp) (Figure 2). The *DDC* gene was not in Hardy Weinberg equilibrium (HWE). It was caused by a higher proportion of GG genotypes in our population, while the AA genotype was rare. The allele and genotype frequencies of the *DDC* gene are shown in Table 2.

### The association of *DDC* gene polymorphism with carcass characteristics, physical properties of lamb, and retail cut of the carcass

The association analysis of the *DDC* gene polymorphisms showed that the *DDC* gene (g.5377439 G>A) was significantly associated (p≤0.05) with carcass characteristics, including carcass percentage, length carcass, hot and cold carcass. Additionally, the *DDC* gene was associated (p≤0.05) with physical properties of lamb quality traits, including pH value (Table 3). The GG genotype was associated with enhanced carcass characteristics, while the AG genotype was associated with improved physical properties of lambs. Furthermore, the *DDC* gene also had a significantly associated (p≤0.05) with all retail cut carcass parameters (Table 4).

### The association of *DDC* gene polymorphism with fatty acid composition

The polymorphisms of the *DDC* gene were significantly associated (p≤0.05) with the fatty acid composition that was fat content; SFA including pentadecanoic acid (C15:0), tricosylic acid (C23:0), and lignoceric acid (C24:0); MUFA including oleic acid (C18:1n9c), elaidic acid (C18:1n9t), and nervonic acid (C24:1); and PUFA including linoleic acid (C18:2n6c), arachidonic acid (C20:4n6), and cervonic acid (C22:6n3). The AA genotype of the *DDC* gene had a lower total fatty acid composition while having a higher value of PUFA (Table 5).

### The association of *DDC* gene polymorphism with mineral content

The *DDC* gene was significantly associated (p≥0.05) with mineral content, potassium (K). The AA genotype had a higher potassium content than the other genotype (Table 6).

### Expression analysis of *DDC* gene

**Figure 3:**
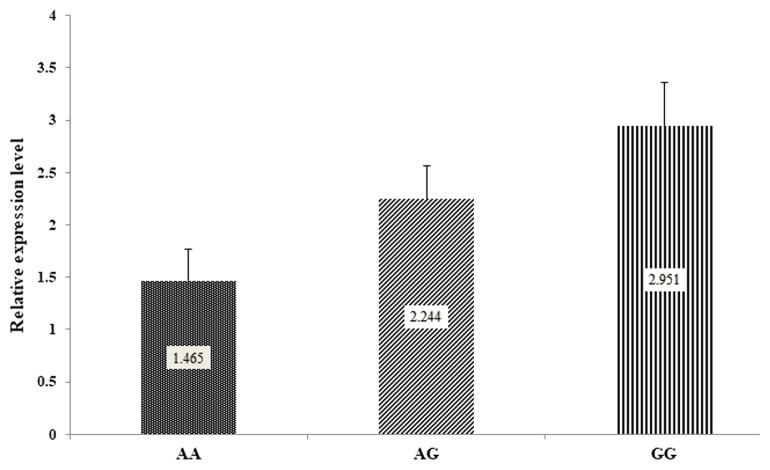
The relative expression level of the *DDC* gene in animals with divergent genotypes for lamb quality traits using qRT-PCR. The genotypes were placed on X-axis. The relative expression level of the gene is represented by the Y-axis. *DDC*, dopa decarboxylase; qRT-PCR, quantitative reverse-transcription-polymerase chain reaction. The mRNA expression was not significantly different between AA, AG, and GG genotypes (p≥0.05).

The result showed that the *DDC* gene had expression transcript in rams with different lamb quality, carcass characteristics, retail meat cut, fatty acid composition, and mineral content. The mRNA of *DDC* gene expression was not significantly different (p≥0.05) between AA, AG, and GG genotypes (Figure 3).

## DISCUSSION

PCR-RFLP successfully amplified the polymorphisms of the *DDC* gene for all our samples of Indonesian sheep. The *DDC* gene had three genotypes (AA, AG, and GG). The GG genotype was dominant, while the AA genotype was relatively rare in the populations. All genotypes were found in each breed, except CAS, in which we did not find the GG genotype. The presence of crosses may influence the missing genotype in the CAS sheep, so the GG genotype experienced selection in the population. The SNP of the *DDC* gene was not in HWE. The DDC g. 5377439 G>A polymorphism was significantly associated (p≤0.05) with lamb quality, including characteristic carcass, physical properties of lamb, retail cut carcass, fatty acid composition, and mineral content. The GG genotype was associated with higher carcass characteristics, while the AG genotype was associated with higher physical properties of lamb. All parameters of characteristic carcasses were significantly different (p≤0.05) except for the bodyweight of the lamb. Bodyweight generally had a positive correlation with the percentage of carcasses. Sheep with a higher body weight will have a higher carcass percentage too. The GG genotype sheep have higher body weight and carcass percentage than the AA and AG genotypes. This result agrees with the study by Yalcintan et al [20] which compares carcass and meat quality characteristics of lambs reared in different seasons. Dressing percentage is highly variable in small ruminants, ranging from 36% to about 60%, and depends on numerous factors, such as age, weight at slaughter, housing system, breed, and sex. Purbowati et al [21] reported that the average dressing percentage of thin-tailed sheep in Indonesia was 40%. Lamb quality parameters were significantly associated (p≤ 0.05) with pH value. The AG genotypes had a lower pH value than the AA and GG genotypes. This study’s pH value is in line with the CL value. This finding follows the result of the study by Utama et al [22]. Lambs with a lower pH value will have a lower CL. Kim et al [23] reported that higher water-holding capacities are owed to higher pH ranges than PSE (pale soft exudative). The meat with lower WHC (low pH) is associated with less water than regular meat.

Retail cut carcass in this study was significantly different (p≤0.05) among AA, AG, and GG genotypes in the *DDC* gene including, leg, loin, flank, shoulder, rack, breast, shank, and neck for some parts of the muscle, bone, subcutaneous fat, IMF, and pelvic fats. The GG genotype had a higher retail carcass cut than the AA and AG genotypes. The result is in line with its carcass weight which is also higher than the others. In terms of size, the main carcass pieces in succession are leg, shoulder, breast, neck, rack, loin, shank, and flank. The primary cuts in lamb carcasses that have the highest value are the shoulder, rib (rack), loin, and leg cuts. However, the lower value cuts are the neck, breast-fore shank, and flank [24]. The totals percent of lean, bone, and fat are 58% to 66%, 19% to 23%, and 4% to 16%, respectively, for sheep with bodyweight 15 to 23 kg [20]. Total subcutaneous fat in this study was higher than IMF, similar to the studies of Yalcintan et al [20]. Furthermore, carcass composition will be changed due to increased slaughter weight, which appears as a decrease in lean ratio and an increase in the fat percentage, especially subcutaneous fat, in the carcass [25–27].

The *DDC* gene polymorphisms were significantly associated (p≤0.05) with fatty acid composition, namely fat content; SFA including pentadecanoic acid (C15:0), tricosylic acid (C23:0), and lignoceric acid (C24:0); MUFA including oleic acid (C18:1n9c), elaidic acid (C18:1n9t), and nervonic acid (C24:1); and PUFA including linoleic acid (C18:2n6c), arachidonic acid (C20:4n6), and cervonic acid (C22:6n3). The AA genotype of the *DDC* gene had a lower total fatty acid composition while having a higher value of PUFA. Generally, the fatty acid composition is dominated by SFA [28–30]. Stearic acid (C18:0) is one of the major SFA besides myristic acid (C14:0) and palmitic acid (C16:0). Senyilmaz-Tiebe et al [31] reported that this fatty acid could decrease cardiovascular and cancer risk. The fatty acid oxidation process will produce acetyl-CoA, the starting molecule for cholesterol synthesis [32]. MUFA and PUFA are unsaturated fatty acids that have benefits for health which could reduce LDL cholesterol (low-density lipoprotein) and increase high-density lipoprotein cholesterol levels. The ratio between PUFA and SFA is an index normally used to assess the impact of diet on cardiovascular health. It hypothesizes that all PUFAs in the diet can depress LDL cholesterol and lower serum cholesterol levels, whereas all SFAs contribute to high serum cholesterol levels. The recommended value is above 0.45. Thus, the higher this ratio, the more positive effect [33]. The *DDC* gene had significantly associated (p≤0.05) with mineral content, potassium (K). The AA genotype had a higher potassium content than the other genotype. Potassium is a shortfall nutrient that is not usually found in fortified foods or commonly consumed as dietary supplements. Potassium plays a role in heart health, hypertension, stroke, cardiovascular disease, bone health, the kidneys, and the transmission of messages through the nervous system [34]. Adequate potassium intake is 3,400 milligrams (mg) per day for healthy adult males and 2,600 mg per day for healthy adult females [35].

Studies in various organisms have shown that the *DDC* gene is involved in regulating the brain [10–12]. In our expression study, the DDC gene was not significantly different (p≥0.05) between AA, AG, and GG genotypes. The DDC encodes a pyridoxal 5′-phosphate-reliant enzyme that facilitates the synthesis of several critical neuroactive BA in the brain. This gene is closely related to serotonin production in the brain. Individuals with a *DDC* gene deficiency will experience decreased serotonin levels which can cause developmental delays and movement disorders [11]. L-dopa decarboxylase (DDC) catalyzes the biosynthesis of bioactive amines, such as dopamine, serotonin, and histidine, which are expressed in the nervous system and peripheral tissues, including the liver [36]. The role of the *DDC* gene in the synthesis of bioactive amines supports that the *DDC* gene (g.5377439 G>A) may contribute to lamb quality including carcass characteristics, physical meat, retail meat cut, fatty acid composition, and mineral content.

## CONCLUSION

The SNP g. 5377439 G>A of the *DDC* gene was found to be polymorphic in Indonesian sheep with genotypes AA, AG, and GG. These variations genotypes of the *DDC* gene were significantly associated with the carcass percentage, length carcass, hot and cold carcass, pH value, retail cut carcass, fat content, pentadecanoic acid, tricosylic acid, lignoceric acid, oleic acid, elaidic acid, nervonic acid, linoleic acid, arachidonic acid, cervonic acid, and potassium content. The GG genotype had the best association with several favorable traits. The polymorphisms of the *DDC* gene might contribute to carcass characteristics, physical properties of lamb, retail cut carcass, fatty acid composition, and mineral content.

## Figures and Tables

**Table 1:** Primer sequences of *DDC* gene for PCR-RFLP and qRT-PCR

Gene name	Accession number	Primer sequence	Application	AT (°C)	Product size (bp)	Restriction enzyme	SNP	Digested fragments length (bp)
*DDC*	NC_019461.2	F: 5′-GCA CCT TCT TAG CCA CTC AT-3′ R: 5′-AGA CAT GTA CTA GGC ACT GC-3′	Genotyping	60	419	*Msp*I (5′-CCGG-3′)	g.5 377 439 G > A	GG: 253 and 166 bp AG: 166, 253, and 419 AA: 419
*DDC*	XM_004002351	F: 5′-ATC CAC TTG GTC CCT TGT TC-3′ R: 5′-CGG AAC TCG CTT GCA TTC AT-3′	qRT-PCR	50	188	-	-	-
*GAPDH*	NC_019460.2	F: 5′-GAG AAA CCT GCC AAG TAT GA-3′ R: 5′-TAC CAG GAA ATG AGC TTG AC-3′	qRT-PCR	60	203	-	-	-
*β-Actin*	NC_019471.2	F: 5′GAA AAC GAG ATG AGA TTG GC-3′ F: 5′CCA TCA TAG AGT GGA GTT CG-3′	qRT-PCR	60	194	-	-	-

*DDC*, dopa decarboxylase; PCR-RFLP, polymerase chain reaction-restriction fragment length polymorphism; qRT-PCR, quantitative real time-polymerase chain reaction; AT, annealing temperature; SNP, single nucleotide polymorphism; GAPDH, = glyceraldehyde-3-phosphate dehydrogenase.

**Table 2:** The number of animals per genotype and allele frequency of each sheep breed

Sheep breed	N	Genotype frequency			Allele frequency		Chi square (χ^2^)
	
		AA	AG	GG	A	G	
JFT	16	0.06 (1)	0.06 (1)	0.88 (14)	0.09	0.91	6.39
JTT	102	0.04 (4)	0.24 (25)	0.72 (73)	0.16	0.83	0.94
GS	20	0.10 (2)	0.05 (1)	0.85 (17)	0.12	0.88	11.90
JS	21	0.05 (1)	0.05 (1)	0.90 (19)	0.07	0.93	8.62
GCS	10	0.30 (3)	0.20 (2)	0.50 (5)	0.40	0.60	3.40
CAS	10	0.60 (6)	0.40 (4)	0.00 (0)	0.80	0.20	0.62
BCS	10	0.30 (3)	0.30 (3)	0.40 (4)	0.45	0.55	1.55
Totals	189	0.07 (14)	0.20 (39)	0.72 (136)	0.18	0.82	16.17

N, number of samples, (..) = number of samples which AA, AG, and GG genotype, *χ*^2^ table = 3.84.

**Table 3:** Association of the *DDC* genes with carcass characteristic and lamb quality in Indonesian sheep

Parameters	The genotype of the *DDC* gene (*χ̄* ±SE mean)		

	AA (n = 10)	AG (n = 31)	GG (n = 109)
Live weight (kg)	21.780±2.150	23.550±1.010	25.784±0.453
Carcass percentage (%)	37.230±1.800^b^	40.987±0.974^ab^	42.230±0.480^a^
Length carcass (cm)	78.610±5.260^ab^	74.450±3.510^a^	66.550±1.310^b^
Hot carcass (kg)	8.005±0.989^b^	9.528±0.525^ab^	10.595±0.274^a^
Cold carcass (kg)	7.620±1.010^b^	9.327±0.546^ab^	10.363±0.278^a^
Tenderness (kg/cm^2^)	3.700±0.437	3.575±0.137	3.659±0.071
pH value	6.457±0.189^a^	5.932±0.073^b^	5.989±0.061^b^
Cooking loss	50.751±0.987	44.630±1.730	46.654±0.730
WHC (mgH_2_0)	82.900±3.080	85.120±2.150	84.200±0.840
WHC (%mgH_2_O)	27.630±1.030	28.375±0.718	28.067±0.280

The numbers shown in parentheses are the number of individuals with the specified genotype.

*DDC*, dopa decarboxylase; ***χ̄***, means of carcass characteristic and lamb quality; SE, standard error; WHC, water holding capacity.

a,b Means with in a row with different letters are different at 5%.

**Table 4:** Association of the *DDC* genes with retail cut carcass of lamb quality

Parameters (g)	The genotype of the *DDC* gene (*χ̄* ±SE mean)		

	AA (n = 5)	AG (n = 27)	GG (n = 68)
Leg	960.0±0.19^b^	1,458.00±0.07^a^	1,596.3±0.04^a^
Muscle	587.00±133.00^b^	970.70±57.90^a^	1,045.90±31.80^a^
Bone	322.20±41.20^b^	384.50±14.60^b^	435.66±9.95^a^
Subcutaneous fats	24.00±1 6.00	58.43±9.57	73.81±8.40
Intramuscular fats	18.82±3.96	28.94±6.16	37.61±5.65
Pelvic fats	6.92±4.90	13.93±3.43	17.20±1.90
Loin	223.4±0.06^b^	346.40±0.02^ab^	397.77±0.02^a^
Muscle	114.70±34.30^b^	213.80±15.40^a^	217.83±9.30^a^
Bone	88.30±21.00^ab^	86.78±6.29^b^	110.72±5.68^a^
Subcutaneous fats	9.56±5.11	28.22±5.30	42.80±4.94
Intramuscular fats	6.82±4.18	10.76±3.90	16.13±3.02
Pelvic fats	14.10±10.10	39.90±12.10	47.98±7.42
Flank	72.00±0.03^b^	120.50±0.01^ab^	154.22±0.01^a^
Muscle	53.90±22.10	89.80±10.40	108.47±6.52
Bone	0.00±0.00	0.00±0.00	0.03±0.03
Subcutaneous fats	10.70±3.12	24.07±2.90	28.63±2.81
Intramuscular fats	3.46±2.16	4.50±1.41	12.91±3.50
Shoulder	468.60±0.07^b^	756.00±0.05^a^	862.60±0.04^a^
Muscle	278.30±54.40^b^	479.90±34.00^a^	546.70±21.20^a^
Bone	145.40±21.20	182.60±10.90	221.84±9.63
Subcutaneous fats	11.28±9.18	32.05±5.55	38.40±4.97
Intramuscular fats	23.14±8.81	46.12±7.61	61.11±6.64
Rack	235.30±0.06	381.30±0.03	408.10±0.02
Muscle	116.10±39.50^b^	222.80±17.50^a^	222.10±9.87^a^
Bone	104.50±13.20	122.53±6.83	132.55±4.78
Subcutaneous fats	6.26±3.86	21.93±4.40	35.11±4.59
Intramuscular fats	3.50±1.98	17.24±4.95	15.84±2.22
Breast	247.00±0.05^b^	408.20±0.03^ab^	473.50±0.02^a^
Muscle	115.50±26.90^b^	213.80±16.70^a^	240.10±9.77^a^
Bone	89.70±11.90^b^	119.74±7.79^ab^	139.46±4.50^a^
Subcutaneous fats	18.86±8.00	29.80±3.72	43.71±4.94
Intramuscular fats	14.78±9.72	36.75±8.20	40.90±4.61
Shank	268.00±0.06	378.40±0.02	389.5±0.01
Muscle	159.40±42.80^b^	250.80±15.00^a^	240.22±7.75^a^
Bone	91.60±11.50	112.25±4.37	117.47±3.04
Subcutaneous Fats	8.56±5.68	17.27±1.71	17.07±1.59
Intramuscular Fats	6.00±1.76	6.52±1.25	9.14±0.91
Neck	290.30±0.08^b^	429.20±0.03^ab^	476.50±0.02^a^
Muscle	166.30±54.50^b^	267.20±19.20^ab^	284.90±10.80^a^
Bone	102.80±21.00	135.80±11.70	143.08±7.68
Subcutaneous fats	13.28±5.77	24.34±8.90	20.98±2.35
Intramuscular fats	2.70±2.70	12.61±2.16	19.78±3.05
Totals	2,741.00±59.00	4,310.00±27.00	4,787.00±17.00

The numbers shown in parentheses are the number of individuals with the specified genotype.

*DDC*, dopa decarboxylase; ***χ̄***, means of retail cut carcass; SE, standard error.

a,b Means with in a row with different letters are different at 5%.

**Table 5:** Association of *DDC* genes with the fatty acid composition in Indonesian sheep

Parameters (%)	The genotype of the *DDC* gene (*χ̄* ±SE mean)		

	AA (n = 9)	AG (n = 31)	GG (n = 110)
Fat content	2.521±0.673^b^	4.917±0.667^ab^	5.824±0.386^a^
Saturated fatty acid	36.520±2.250	42.200±1.370	40.850±0.808
Caprylic acid, C8:0	0.000±0.000	0.081±0.034	0.115±0.354
Capric acid, C10:0	0.052±0.014	0.093±0.013	0.355±0.164
Lauric acid, C12:0	0.482±0.138	0.403±0.067	0.335±0.042
Tridecylic acid, C13:0	0.009±0.004	0.015±0.004	0.072±0.043
Myristic acid, C14:0	2.349±0.429	2.714±0.256	2.620±0.141
Pentadecylic acid, C15:0	0.360±0.047^b^	0.499±0.036^ab^	0.539±0.017^a^
Palmitic acid, C16:0	16.300±1.990	18.073±0.837	18.739±0.438
Margaric acid, C17:0	0.592±0.081	0.770±0.064	0.898±0.038
Stearic acid, C18:0	15.830±1.750	18.028±0.810	15.802±0.492
Arachidic acid, C20:0	0.196±0.049	1.170±0.296	1.088±0.147
Heneicosylic acid, C21:0	0.039±0.006	0.188±0.051	0.145±0.024
Behenic acid, C22:0	0.132±0.045	0.068±0.014	0.087±0.041
Tricosylic acid, C23:0	0.064±0.024^a^	0.043±0.009^ab^	0.025±0.004^b^
Lignoceric acid, C24:0	0.111±0.058^a^	0.049±0.016^ab^	0.025±0.006^b^
Unsaturated fatty acid	31.880±4.480	33.840±1.190	36.468±0.649
Monounsaturated fatty acid	24.110±3.800^b^	30.340±1.390^ab^	33.022±0.709^a^
Myristoleic acid, C14:1	0.169±0.047	0.186±0.056	0.129±0.009
Palmitoleic acid, C16:1	16.300±1.990	18.739±0.438	1.483±0.055
Ginkgoleic acid, C17:1	0.352±0.077	0.333±0.048	0.943±0.280
Oleic acid, C18:1n9c	21.510±3.830^ab^	21.400±2.050^b^	27.173±0.827^a^
Elaidic acid, C18:1n9t	0.692±0.458^b^	0.693±1.700^a^	0.311±0.507^b^
Paullinic acid, C20:1	0.186±0.101	0.104±0.045	0.111±0.023
Erucic acid, C22:1n9	0.002±0.002	0.022±0.008	0.043±0.016
Nervonic acid, C24:1	0.097±0.069^a^	0.047±0.013^ab^	0.028±0.005^b^
Polyunsaturated fatty acid	7.770±1.720^a^	3.507±0.430^b^	3.446±0.203^b^
Linoleic acid, C18:2n6c	5.010±1.440^a^	1.515±0.259^b^	1.725±0.135^b^
y-Linolenic acid, C18:3n6	0.056±0.036	0.064±0.011	0.065±0.008
α-Linolenic acid, C18:3n3	0.288±0.071	0.283±0.049	0.367±0.027
Eicosadienoic acid, C20:2	0.106±0.051	0.041±0.006	0.069±0.025
Dihomo-y-linolenic acid, C20:3n6	0.208±0.079	0.071±0.015	0.074±0.022
Arachidonic acid, C20:4n6	1.660±0.512^a^	0.998±0.221^ab^	0.642±0.096^b^
Docosadienoic acid, C22:2	0.000±0.000	0.019±0.011	0.049±0.016
Eicosapentaenoic acid, C20:5n3	0.283±0.081	0.275±0.049	0.280±0.032
Cervonic acid, C22:6n3	0.121±0.062^a^	0.039±0.007^b^	0.047±0.006^b^
Fatty acid total	68.420±5.620^b^	77.030±1.830^ab^	77.870±0.877^a^

*DDC*, dopa decarboxylase; ***χ̄***, means of fatty acid composition; SE, standard error.

The numbers shown in parentheses are the number of individuals with the specified genotype.

a,b Means with in a row with different letters are different at 5%.

**Table 6:** Association of *DDC* gene with mineral content of lamb quality

Parameters (mg/kg)	The genotype of the *DDC* gene (*χ̄* ±SE mean)		

	AA (n = 2)	AG (n = 18)	GG (n = 80)
Iron (Fe)	16.09±6.55	18.28±1.76	18.85±0.90
Zinc (Zn)	28.40±10.20	26.34±2.29	26.06±1.10
Potassium (K)	3,863.00±221.00^a^	3,054.00±179.00^ab^	2,641.90±95.30^b^
Selenium (Se)	6.60±1.43	5.14±0.84	6.39±0.33

The numbers shown in parentheses are the number of individuals with the specified genotype.

*DDC*, dopa decarboxylase; ***χ̄***, means of mineral content; SE, standard error.

a,b Means with in a row with different letters are different at 5%.
